# Ravulizumab in NMOSD with associated interstitial pneumonitis in a 59-year-old female patient: a case report

**DOI:** 10.3389/fimmu.2025.1671145

**Published:** 2026-01-05

**Authors:** Andrea Neundorf, Ralf Dittrich

**Affiliations:** Niels-Stensen-Kliniken, Marienhospital Osnabrück, Osnabrück, Germany

**Keywords:** neuromyelitis optica spectrum disease, ravulizumab, exacerbation, case report, interstitial pneumonitis

## Abstract

**Introduction:**

Neuromyelitis optica spectrum disorder (NMOSD) is an autoimmune disease of the central nervous system often associated with aquaporin-4-immunoglobulin-G (AQP4-IgG), which activate the complement system, and it can lead to progressive neurological disability. Complement inhibitors have been shown to be effective in preventing relapses. While there exist rare case reports on the early initiation of eculizumab in the acute exacerbation phase, no data are available for early treatment with ravulizumab.

**Case:**

A 59-year-old patient was diagnosed with AQP4-IgG seropositive NMOSD upon presentation with paraplegia, and MRI-confirmed longitudinally extensive transverse myelitis from C2 to T5 in November 2023. Treatment included high-dose methylprednisolone, followed by immunoadsorption (IA) and complement-inhibition therapy with ravulizumab initiated 13 days after attack onset alongside with IA. The patient additionally developed an interstitial pneumonia complicating the case, likely attributable to complement-mediated autoimmune processes. Over time, there was significant improvement in neurological and respiratory conditions, as evidenced by reduced spinal cord edema and partial resolution of pulmonary infiltrates on follow-up imaging in January 2024. By January 2025, the patient exhibited further neurological improvement and regained the ability to stand upright with external support. Ravulizumab therapy was well tolerated, and the patient will continue with eight-weekly infusions.

**Conclusion:**

Overall, this case highlights that early targeted immunotherapy with ravulizumab within two weeks of attack onset, supported by IA, can be an effective choice for controlling NMOSD disease activity and improving patient outcomes. NMOSD-related interstitial pneumonitis, though rare, underscores the need for vigilance in recognizing atypical manifestations of the disease.

## 1 Introduction

Neuromyelitis optica spectrum disorder (NMOSD) is an autoimmune neuroinflammatory disease of the central nervous system associated with aquaporin-4-immunoglobulin-G (AQP4-IgG). In AQP4-IgG–positive NMOSD, pathogenic autoantibodies bind to aquaporin-4 on astrocytes, causing complement cascade activation and formation of the membrane attack complex (C5b-9). This process induces astrocyte injury and secondary demyelination (1). Recurrent myelitis and optic neuritis attacks usually lead to progressive neurological disability (2). Rare cases of organ manifestations, e.g., pulmonary infiltrations, have been reported (3–5).

Acute treatment involves high-dose glucocorticoid with or without apheresis therapy, i.e., plasma exchange or immunoadsorption (IA) followed by an oral glucocorticoid tapering regimen. Long-term immunotherapy should be initiated after or during acute treatment to prevent relapses, as these are mostly associated with worsening disability. In this context, the preferred treatment options include complement inhibition with eculizumab or ravulizumab, B-cell depletion with rituximab (off-label for NMOSD in Germany and other countries) or inebilizumab, or IL-6 blockade with satralizumab. Classical immunosuppressants (i.e., azathioprine, mycophenolate mofetil) or IL-6 blockade with tocilizumab remain second-line options for long-term relapse prevention (6).

Both ravulizumab and eculizumab are directed against complement component C5 and prevent its cleavage and subsequent formation of C5b-9. Thus both provide equivalent mechanisms of complement blockade and protect astrocytes from complement-mediated cytotoxicity (1). Ravulizumab, however, enables maintenance dosing every eight weeks compared to the biweekly schedule required for eculizumab (1). The safety and efficacy of the C5 complement inhibitor ravulizumab for the prevention of relapses in adult patients with AQP4-IgG seropositive NMOSD were evaluated in a phase 3, open-label, multinational, multicenter clinical trial (CHAMPION-NMOSD) (7). In this study, ravulizumab significantly reduced the risk of relapse by 98.6% compared to a historical placebo control from the pivotal eculizumab trial (8). While rare case reports on the initiation of eculizumab in the acute exacerbation phase exist, no data are available for early treatment with ravulizumab. The present report delineates a case of seropositive NMOSD presenting with longitudinally extensive transverse myelitis and pulmonary involvement with initiation of ravulizumab in the acute exacerbation phase after first diagnosis of NMOSD in the treated patient.

## 2 Case description

The patient, a 59-year-old female, was initially admitted for postoperative care following a left knee total endoprosthesis. On October 30, 2023, she was emergently transferred due to acute abdominal pain. Laboratory tests revealed elevated D-dimer levels, and further diagnostics identified moderate chronic and moderate active *Helicobacter pylori*-associated antrum and corpus gastritis (**Table 1**).

**Table 1:** Medical history, secondary diagnoses and concomitant medication.

• Post-traumatic knee osteoarthritis (no current treatment)
• Left knee total endoprosthesis (no current treatment)
• Bony-consolidated proximal tibial osteotomy on the left (no current treatment)
• Prior Tako Tsubo cardiomyopathy (no current treatment)
• Benign essential hypertension (treated with bisoprolol and torasemide)
• One-vessel coronary artery disease (treated with acetylsalicylic acid and atorvastatin)
• Prior tuberculosis (no current treatment)
• IgA nephritis (no current treatment)
• Diabetes mellitus type 2 (treated with metformin and empagliflozin)
• Helicobacter-associated antrum and corpus gastritis (treated with pantoprazole)
• Small axial hernia (no current treatment)
• Hypochromic microcytic iron-deficiency anemia (treated with oral iron supplementation)
• Further medication: Pipamperone, potassium supplementation during hospital phase

**Figure 1:**
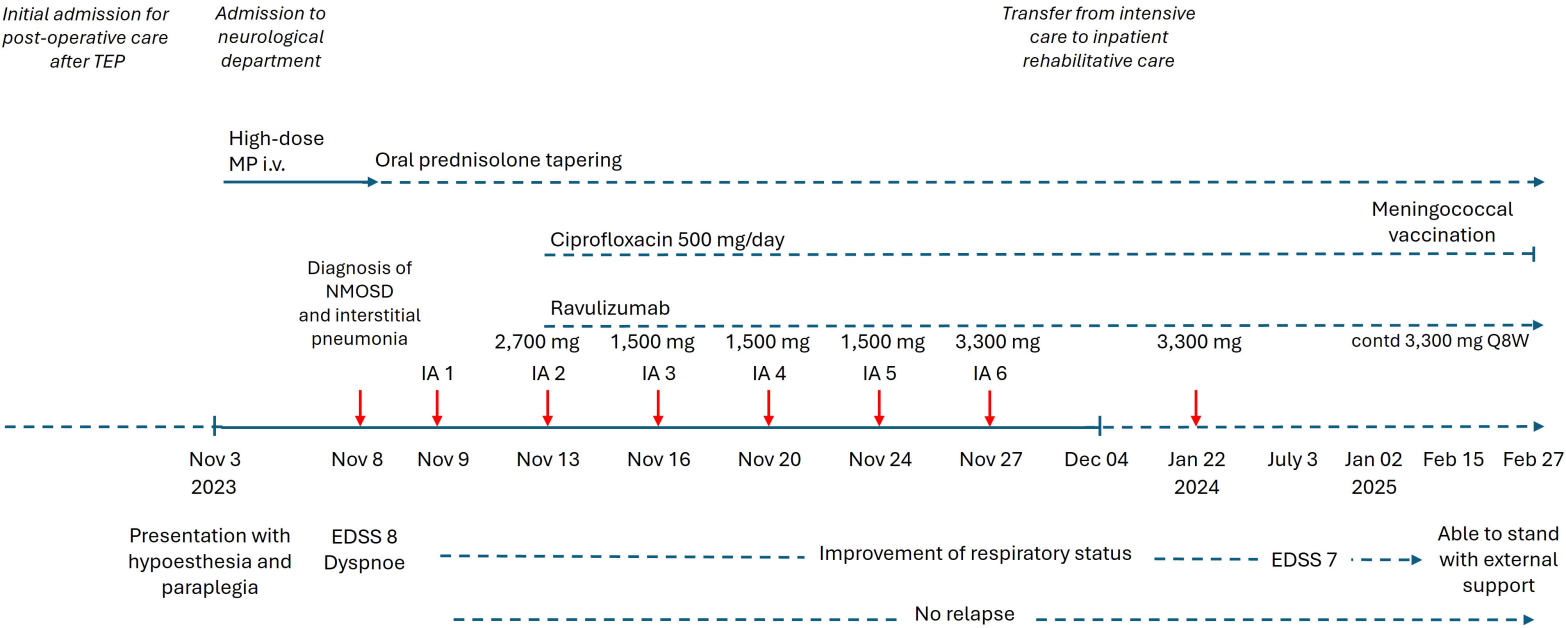
Timeline of clinical course, acute therapy during hospital admission and follow-up.

**Figure 2:**
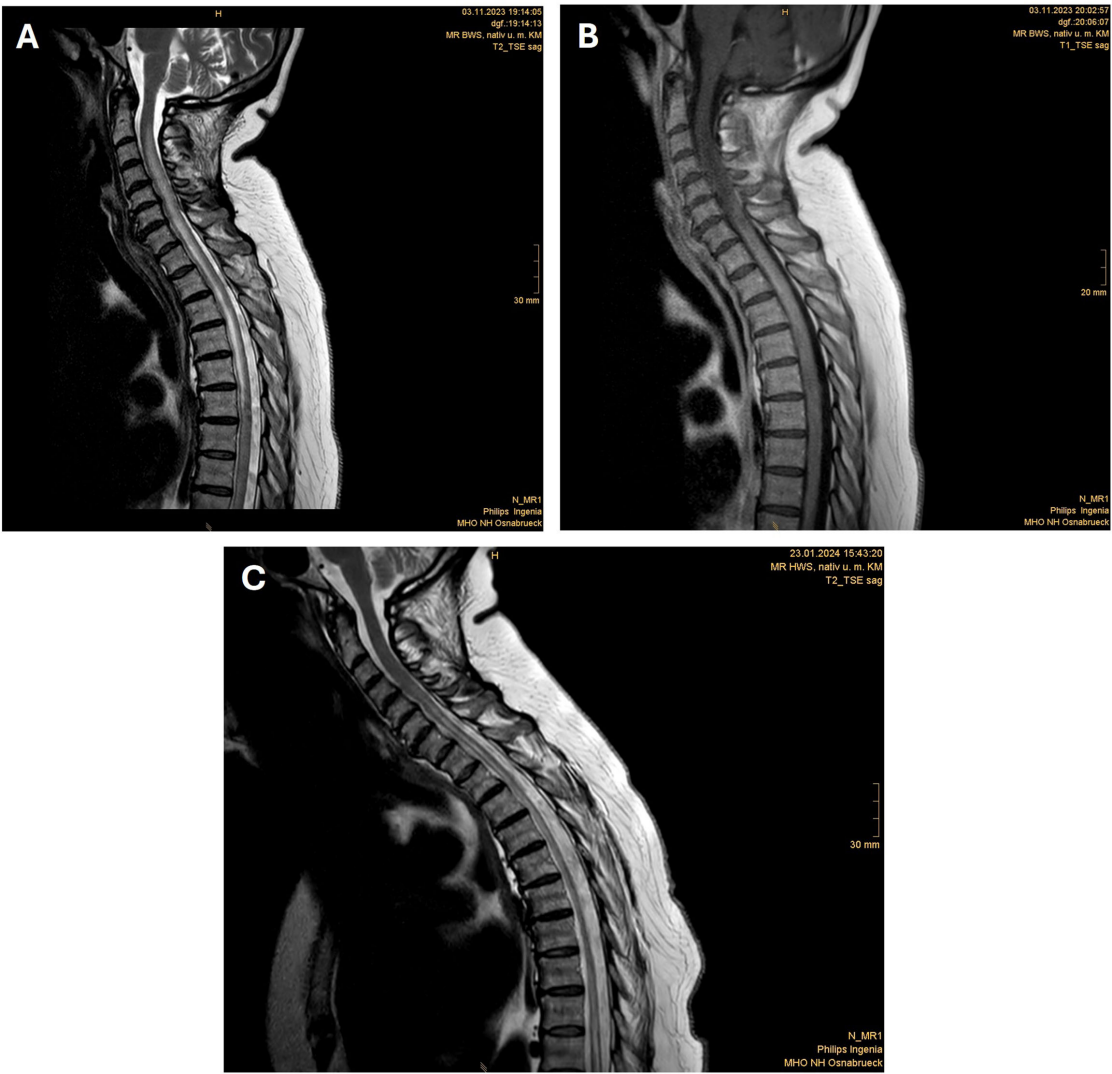
Spine MRI shows longitudinally extensive transverse myelitis from C2 to T5 before ravulizumab initiation in T2W **(A)** and T1W with gadolinium enhancement **(B)** and regression of the spinal cord edema but persistent longitudinal atrophy after ravulizumab initiation in T2W **(C)**.

On the night of November 2, 2023, the patient exhibited progressive hypoesthesia below thoracic level 5 (TH5) and paraplegia of the legs (**Figure 1**) and bowel-bladder dysfunction with incontinence. Upon admission due to these symptoms to our department, a neurological examination confirmed incomplete paraparesis syndrome from TH5 with hypoesthesia from TH5 and muscle strength of 1–2/5 in both legs, accompanied by bilateral positive Babinski signs. The patient also exhibited bladder bowel dysfunction. Gadolinium-enhanced magnetic resonance imaging (MRI) of the cervical, thoracic, and lumbar spine revealed a longitudinally extensive transverse myelitis from C2 to T5 (**Figure 2**). Lumbar puncture findings included cell counts of 25 cells/μL and elevated protein (0.86 g/L). The Expanded Disability Status Scale (EDSS) score was 8. On November 8, 2023, serological analysis revealed a high AQP4-IgG titer (1:1,280), confirming the diagnosis of NMOSD. Differential diagnostics for infectious diseases or autoimmune diseases were performed, including systemic autoimmune conditions (e.g., systemic lupus erythematosus), but were unremarkable. MOG antibodies, oligoclonal bands, and MRZR (measles, rubella, and varicella zoster virus reaction) were all negative.

The initial empirical treatment administered upon presentation included intravenous ceftriaxone, acyclovir, and high-dose methylprednisolone. The latter was administered from November 3 to November 8, 2023, with a regimen of 1,000 mg/day for two days, 2,000 mg/day for three days, followed by tapering of oral prednisolone starting with a dose of 1 mg/kg bodyweight (**Figure 1**). Antibiotics and antivirals were discontinued as soon as a diagnosis of NMOSD was confirmed and infectious causes excluded.

In addition to the high-dose methylprednisolone regimen, a complement-binding antibody therapy with ravulizumab and IA was initiated. Seven IA sessions were performed between November 9 and 27, 2023. The initiation of treatment was delayed until day 4 as it was necessary to discontinue ACE inhibitor treatment to prevent activation of the bradykinin system during IA. Despite discontinuation of ACE inhibitor treatment, bradykinin activation-associated hypotension occurred, which necessitated i.v. catecholamine support. The patient exhibited subsequent instances of sporadic hemodynamic instability, which again necessitated the administration of catecholamines, but which did not result in adverse outcomes. These phases of circulatory dysregulation also occurred on days without IA, but in close temporal association. Repeated central imaging to clarify hypothalamic disorders revealed no intracerebral lesions and in particular no lesions in the circulatory regulatory centers were identified.

Ravulizumab therapy was initiated on November 15, 2023 (2,700 mg), followed by a second dose on November 27, 2023 (3,300 mg), with planned continuation every eight weeks (Q8W). In addition, ravulizumab was readministered directly after the IA sessions (1,500 mg) according to the recommendations (9). Meningococcal vaccination was postponed as the acuity of this NMOSD case needed immediate therapeutic intervention. Meningococcal prophylaxis with ciprofloxacin (500 mg/day) was initiated in conjunction with ravulizumab (**Figure 1**). Vaccination status according to national recommendations was up-to-date, and no other vaccinations were indicated. The patient received in addition oxycodone/naloxone, melatonin at night, and duloxetine for treatment of severe neuropathic pain in the extremities and back, which yielded significant pain relief over time.

**Figure 3:**
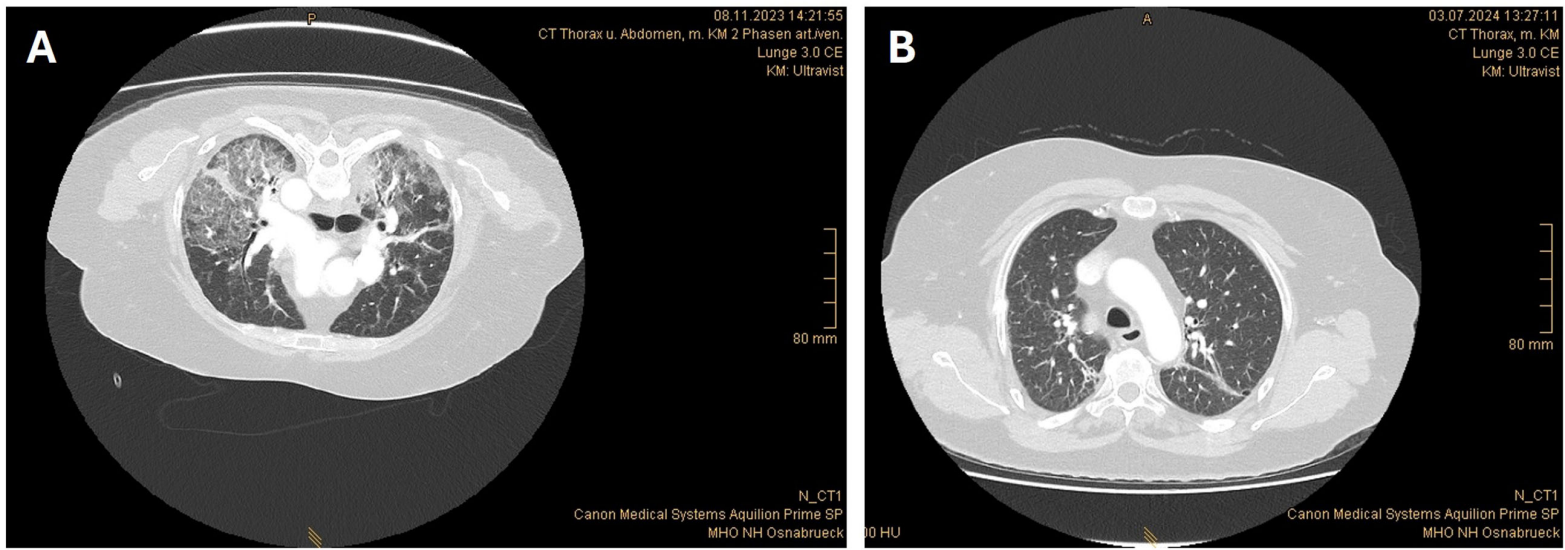
Thorax CT shows bilateral extensive interstitial pneumonia before ravulizumab initiation **(A)** and reduced pulmonary infiltrates, residual consolidation from resolved pneumonitis, as well as suspected encapsulated effusions after ravulizumab initiation **(B)**.

During hospitalization, the patient also developed an oxygenation disorder. Thoracic computed tomography (CT) revealed multifocal areas of consolidation and ground-glass opacification indicative of bilateral extensive interstitial pneumonia (**Figure 3**). This finding aligns with reports of rare cases of complement-mediated autoimmune lung infiltrations in the context of NMOSD (3–5). The respiratory status of the patient improved under high-flow oxygen therapy and ravulizumab in combination with IA. Subsequent thoracic CT scans revealed reduced pulmonary infiltrates. An additional brain MRI did not reveal any inflammatory lesions or other pathological findings.

On December 04, 2023, the patient was transferred from the intensive care unit to inpatient neurological rehabilitative care. Ravulizumab was continued at 3,300 mg every eight weeks. At the follow-up visit on January 22, 2024, the patient’s clinical status demonstrated improvement. She received a third dose of ravulizumab (3,300 mg). Concurrently, oral prednisolone had already been tapered to 60 mg/day. MRI revealed a significant regression of the spinal cord edema, but persistent longitudinal atrophy (**Figure 2**). Pulmonary findings from the follow-up thoracic CT included reduced pulmonary infiltrates, residual consolidation from resolved pneumonitis, and suspected encapsulated effusions (**Figure 3**).

On July 3, 2024, when the patient presented for re-infusion of ravulizumab, an assessment revealed further neurological improvement, with an EDSS score reduced to 7. Motor strength in the left leg improved, allowing active movement against gravity, though standing ability had not yet been regained. A recent MRI evaluation revealed stable NMOSD-related myelitis, with no new lesions identified. By the most recent assessment on January 02, 2025, the patient had improved further and was able to stand with external support. A request has been made for a subsequent neuro-rehabilitation program to consolidate the progress made.

Ravulizumab therapy was well tolerated, with no reported relapses up to February 27, 2025. The plan is to continue infusions at eight-week intervals, while tapering oral prednisolone by 5 mg/month down to 10 mg/day, with further reductions based on clinical stability. The patient is also receiving vitamin D and calcium for osteoporosis prophylaxis, and a meningococcal vaccination was applied mid-February 2025. Antibiotic prophylaxis was stopped 14 days after vaccination.

### 2.1 Patient Perspective

The patient reported that since starting treatment with ravulizumab, she has experienced a clear improvement in her symptoms. When asked about side effects, the patient mentioned headaches and occasional nausea. The infusion itself is not felt as a burden. However, she emphasized that traveling to the clinic remains extremely difficult and poses a significant challenge.

## 3 Discussion

To the best of our knowledge, this is the first report of early initiation of ravulizumab within two weeks of attack onset (i.e., one week after diagnosis) in a patient with NMOSD, as well as the first report of regression of NMOSD-associated lung involvement through complement inhibition with ravulizumab. Although further confirmation is required, our observations are supported by literature data on complement inhibition in NMOSD. The therapeutic intervention specifically prevents astrocyte damage by blocking C5 cleavage and the subsequent formation of the membrane attack complex. As AQP4 is found also in peripheral tissues, autoantibody-induced complement activation could be involved in manifestations beyond the central nervous system (1). Consequently, inhibiting C5 might alleviate NMOSD-associated lung injury, as illustrated in the present case of interstitial pneumonitis.

Ravulizumab has previously demonstrated a rapid onset of action, exhibiting nearly complete complement inhibition following the initial infusion (10). The clinical efficacy of ravulizumab in the prevention of relapses in adult patients diagnosed with AQP4-IgG seropositive NMOSD was investigated in the CHAMPION-NMOSD study. During the core phase, which spanned 50 weeks, no relapses occurred among patients treated with ravulizumab (7). An interim analysis of the extension phase of the CHAMPION-NMOSD trial, which spanned up to 233 weeks, further corroborated the absence of relapses in all patients over the long term. This finding is accompanied by a statistically significant 98.9% reduction in the risk of relapse when compared to the historical placebo group from the eculizumab pivotal trial. Furthermore, 89.7% of patients exhibited no clinically significant deterioration in EDSS, which substantiates the efficacy of ravulizumab in preserving neurological function (11). The case under consideration aligns with the clinical trial data. Consequently, ravulizumab was able to prevent any relapses over a follow-up period of 15 months after initiation. The present case exhibited a severe attack accompanied by pulmonary involvement, requiring intensive care with high-flow oxygen supplementation. Consequently, we opted to initiate complement inhibition early within two weeks of the onset of the acute attack in conjunction with IA.

However, although IA and ravulizumab were initiated during the acute phase, it might ideally be anticipated earlier, given the rapidity of complement-mediated damage and the fact that a short delay to IA has been reported to be the strongest predictor of clinical outcomes (12). Notably, the present patient had only partially recovered and severe residual neurological deficit (EDSS 7).

It should be noted that hypotension, as observed in the present case during the acute phase, may also result from involvement of cervical descending autonomic pathways, as described previously (13). In the present patient, the episodes of hypotension occurred in close temporal association with the immunoadsorption sessions, and no lesions were detected in central autonomic regulatory regions. However, MRI of the brainstem is subject to technical limitations, and therefore subtle involvement of descending sympathetic pathways cannot be definitively excluded. The possibility that severe NMOSD attacks can induce hemodynamic instability underscores the importance of prompt initiation of high-efficacy immunotherapy.

Case reports on eculizumab in analogous cases of relapse treatment have suggested the effectiveness of early initiation. For instance, Watanabe et al. documented the utilization of eculizumab in a case series of acute NMOSD. The study detailed the cases of five patients, three with optic neuritis, one with myelitis, and one with brainstem encephalitis and myelitis. Despite initial treatment with intravenous methylprednisolone and plasma exchange, symptom relief was insufficient. The administration of eculizumab, initiated between 35 and 61 days after the onset of symptoms, resulted in partial improvement in all cases (14). The report of a severe case involved a 47-year-old woman with seropositive NMOSD who experienced a life-threatening relapse two months after cyclophosphamide treatment, reaching an EDSS score of 9.5 at peak severity. When symptoms did not respond to steroids or plasma exchange, eculizumab was introduced, resulting in rapid improvement in mental status and gradual recovery of motor and brainstem functions (15). In another case, a 12-year-old male patient experienced severe refractory brainstem syndrome due to an NMOSD attack, resulting in respiratory failure that required intubation and ventilation. Despite administration of high-dose steroids and plasma exchange, the patient’s condition deteriorated. Eculizumab was initiated after the final plasma exchange session. Within five days of treatment, symptoms stabilized and began improving, with near full recovery of muscle strength and independent walking achieved three weeks later (16). A 10-year-old girl with extensive transverse myelitis, attributed to seropositive NMOSD, initially received steroids and plasma exchange starting on day 2. On day 12, eculizumab was introduced, resulting in initial benefits. After eight weeks, the patient transitioned to rituximab for a more convenient dosing schedule. However, a relapse occurred ten weeks later, necessitating further plasma exchange and steroids. Eculizumab treatment was subsequently resumed, with plans for a transition to ravulizumab (17). Chatterton et al. described a relapse in a 46-year-old woman presenting with acute monocular vision loss. Treatment with steroids and plasmapheresis over five days failed to halt clinical deterioration. The patient was administered eculizumab two days after the final plasma exchange, resulting in substantial vision improvement. However, after three months the patient had not yet completely recovered. The patient continued to receive eculizumab treatment (18).

In the present case, ravulizumab was selected over eculizumab due to its less frequent infusion schedule. Eculizumab requires administration every two weeks (14) compared to eight weeks for ravulizumab (15). The patient must travel a considerable distance to attend her infusion appointments and, due to her disability, requires an accompanied ambulance transport for each appointment. Consequently, the ravulizumab dosing intervals were deemed more convenient and pragmatic for the patient.

To the best of our knowledge, there have been no documented cases of early treatment initiation for ravulizumab, i.e., within the first few weeks of treatment of the acute attack. The present report is the inaugural documentation supporting the initiation of ravulizumab immediately after the start of acute therapy with steroids and plasmapheresis, providing early, highly efficient broad immunosuppression in case of an acute attack and in the treatment of complement-associated comorbidities or organ involvement. Notably, strong neurological improvement was observed at the initial follow-up visit. The severity of the attack necessitated immediate initiation of complement inhibition, despite the absence of meningococcal vaccination. In general, it is recommended that individuals receive meningococcal vaccination at least two weeks prior to initiation of complement inhibitor therapy. However, it is important to note that meningococcal vaccination can potentially increase the risk of NMOSD attacks for two weeks following vaccination. For NMOSD patients, especially in case of severe illness, immediate initiation of complement inhibitor treatment under antibiotic prophylaxis may be preferable to prior vaccination, taking into consideration the timing of the vaccination and the potential risks. Of note, antibiotic prophylaxis must be continued until at least two weeks after completion of vaccination (6, 9, 19).

With regard to the pulmonary infiltrates in our case, partial resolution was documented in the follow-up visit, further substantiating the efficacy of prompt initiation of complement inhibition in severe cases of NMOSD accompanied by organ manifestations. It is also noteworthy that lung involvement is rarely observed in NMOSD, despite the widespread expression of AQP4 in lung tissue (20). A review of the literature on pulmonary lesions in NMOSD cases has been conducted by Asato et al. (4) These lesions were predominantly characterized as a component of other collagen vascular diseases that co-occurred with NMOSD or as occurrences coinciding with the transient elevation of creatine kinase (CK) in the early stages of the disease (4). Notably, Asato et al. reported a case of seropositive NMOSD accompanied by transient unclassifiable interstitial pneumonia, characterized by elevated serum concentrations of CK and brainstem symptoms (4). The case described by Asato et al. exhibited a degree of similarity with the pediatric case reported by Enriquez, who documented severe brainstem syndrome and respiratory failure (16). However, the case report by Enriquez et al. did not include further information on the pulmonary manifestation. In contrast, Lai et al. presented three cases of NMOSD with organizing pneumonia at the onset of NMOSD. Lung involvement had resolved spontaneously in one patient and after immunotherapy in the other two patients (3). Lai et al. hypothesize that the temporal association of organizing pneumonia with the onset of AQP4-IgG seropositive NMOSD in their patients suggests that the lung, muscle, and neurological involvement might be a single disease entity induced by AQP4-IgG autoimmunity. This assumption is corroborated by Furube et al., who in an immunohistochemical evaluation of lung biopsy specimens from an NMOSD patient with organizing pneumonia found inflammatory debris within affected alveoli. Furthermore, alveolar epithelial cells surrounding the organizing pneumonia lesions exhibited diminished AQP4 expression levels, increased IgG binding, and complement membrane attack complex deposits (5). Nevertheless, the role of AQP4-IgG-associated complement activation in peripheral tissues remains a matter of debate. While evidence from rodent models suggests that AQP4-IgG binding to AQP4 receptors occurs in peripheral tissues, including the trachea, downstream complement-dependent cytotoxicity has not been observed in these models (21). This discrepancy could be attributed to the presence of complement inhibitors, such as CD59, in peripheral tissues (22, 23). It could be postulated that complement-induced damage to peripheral organ tissue in NMOSD manifests only in patients with alterations in CD59 expression or activity. However, the available evidence on this subject in the context of NMOSD organ manifestations remains inconclusive. Our conclusions regarding pulmonary involvement remain limited by the absence of a lung biopsy to definitively confirm immune-complex–mediated tissue injury. Moreover, it cannot be excluded that low-dose corticosteroids contributed to the improvement of pulmonary infiltrates alongside the effects of ravulizumab.

Finally, and for completeness, it should be noted that the patient’s pre-existing IgA nephropathy is not considered part of the NMOSD spectrum. Despite the high expression of AQP4 in renal tissue, there is no clear evidence that AQP4-IgG drives primary glomerular disease (24), and in this case the nephropathy was clinically stable. It is therefore interpreted as an unrelated comorbidity.

Overall, this case underscores the efficacy of early targeted immunotherapy with ravulizumab, supported by IA, in controlling disease activity and enhancing patient outcomes. While NMOSD-related interstitial pneumonitis is a rare occurrence, the present case highlights the need for vigilance in recognizing atypical manifestations of the disease. Prospective randomized studies are essential to ascertain the efficacy of complement inhibition in the treatment of acute attacks in NMOSD.

## Data Availability

The datasets presented in this article are not readily available because of ethical and privacy restrictions. Requests to access the datasets should be directed to the corresponding author/s.
